# Sirolimus effects on cancer incidence after kidney transplantation: a meta-analysis

**DOI:** 10.1002/cam4.487

**Published:** 2015-06-24

**Authors:** Elizabeth L Yanik, Kulsoom Siddiqui, Eric A Engels

**Affiliations:** 1 Division of Cancer Epidemiology and Genetics, National Cancer InstituteBethesda, Maryland; 2 Brown School of Social Work, Washington University in St. LouisSt. Louis, Missouri

**Keywords:** Immunosuppressants, kidney cancer, kidney transplantation, prostate cancer, rapamycin, sirolimus, skin cancer

## Abstract

Sirolimus, an immunosuppressant option for kidney transplant recipients, may reduce cancer risk by interrupting the mammalian target of rapamycin pathway. However, studies of sirolimus and cancer incidence in kidney recipients have not been definitive, and have had limited ability to examine specific cancer types. The literature was systematically reviewed to identify randomized controlled trials (RCTs) and observational studies of kidney recipients that compared sirolimus users to sirolimus nonusers. Meta-analytic methods were used to obtain pooled estimates of the association between sirolimus use and incidence of total cancer and specific cancer types. Estimates were stratified by study type (RCT vs. observational) and use of cyclosporine (an immunosuppressant that affects DNA repair). Twenty RCTs and two observational studies were eligible for meta-analysis, including 39,039 kidney recipients overall. Sirolimus use was associated with lower overall cancer incidence (incidence rate ratio [IRR] = 0.71, 95% CI = 0.56–0.90), driven by a reduction in incidence of nonmelanoma skin cancer (NMSC, IRR = 0.49, 95% CI = 0.32–0.76). The protective effect of sirolimus on NMSC risk was most notable in studies comparing sirolimus against cyclosporine (IRR = 0.19, 95% CI = 0.04–0.84). After excluding NMSCs, there was no overall association between sirolimus and incidence of other cancers (IRR = 1.06, 95% CI = 0.69–1.63). However, sirolimus use had associations with lower kidney cancer incidence (IRR = 0.40, 95% CI = 0.20–0.81), and higher prostate cancer incidence (IRR = 1.85, 95% CI = 1.17–2.91). Among kidney recipients, sirolimus users have lower NMSC risk, which may be partly due to removal of cyclosporine. Sirolimus may also reduce kidney cancer risk but did not appear protective for other cancers, and it may actually increase prostate cancer risk.

## Introduction

In 2013, more than 16,000 individuals with end-stage renal disease received kidney transplants in the United States 1. After transplantation, immunosuppressant medications are administered to avert rejection of the transplanted kidney 2. One consequence of this immunosuppression is an elevated risk of cancer, especially for infection-related cancers and nonmelanoma skin cancer (NMSC, the most common cancer type) 3,4. This also translates into elevated cancer-related mortality 5.

Of interest, one class of immunosuppressants, mammalian target of rapamycin (mTOR) inhibitors, may have anticarcinogenic effects 6. These medications target and inactivate the mTOR protein kinase, a key regulator of the cell cycle 7. Inhibiting the mTOR pathway interferes with cell growth and proliferation, and suppresses normal immune responses by disrupting T-cell proliferation 2,8. In malignancies, the mTOR pathway is often hyperactivated, and the use of mTOR inhibitors can slow the proliferation of malignant cells and interfere with angiogenesis needed for tumor growth 7,9. mTOR inhibitors can be used to treat cancer, particularly renal cell carcinoma and Kaposi sarcoma (KS) 10–12.

In 1999, sirolimus became the first mTOR inhibitor approved for use as an immunosuppressant in kidney transplant recipients by the U.S. Food and Drug Administration, and it remains the most commonly used mTOR inhibitor in kidney recipients in the United States 8. There is some concern that sirolimus may be associated with poor long-term kidney function and a higher risk of death 13–16. On the other hand, given the mechanistic understanding of the mTOR pathway and the evidence for mTOR inhibitor efficacy in cancer treatment, it is hypothesized that sirolimus may reduce cancer incidence in kidney recipients.

Currently available data on sirolimus and cancer incidence in kidney recipients have not appeared to be definitive. Because most cancer outcomes are somewhat uncommon, small randomized controlled trials (RCTs) evaluating sirolimus-based immunosuppressant regimens have not been able to assess cancer associations. Meanwhile, results from larger observational studies have not been conclusive 17,18. Moreover, prior meta-analyses have aimed to discern the effect of sirolimus on total cancer risk 13,14 or on NMSC in particular 19, but little is known about the effects of sirolimus on other specific cancer types.

Understanding the cancer-specific effects of sirolimus would help with understanding the etiologic role of the mTOR pathway, and would also inform decisions about immunosuppressant use for recipients with known cancer-risk factors, for whom the advantages of sirolimus use might outweigh possible disadvantages. Therefore, we conducted a systematic review and meta-analysis using results from RCTs and observational studies to determine the effect of sirolimus on overall and type-specific cancer incidence.

## Methods

### Literature search

A PubMed literature search updated through July 2014 was conducted to identify studies that compared sirolimus to other immunosuppressants in adult kidney transplant recipients and had information on cancer risk. Specific PubMed search terms were “sirolimus AND kidney transplants”, as well as “rapamycin AND kidney transplants.” We only included studies that compared a group of individuals exposed to sirolimus to a group unexposed to sirolimus. Both RCTs and observational studies were included. We excluded case reports, case series, studies that included nonkidney organ recipients or pediatric recipients, and studies of the mTOR inhibitor everolimus. After identifying an initial pool of articles, we reviewed the bibliographies of the selected articles and prior meta-analyses, and used Web of Science to search citations of the articles, to identify additional studies for inclusion.

Initially, RCTs comparing sirolimus to other immunosuppressants were eligible even if cancer outcomes were not reported in the article. If cancer results were not listed, we contacted the corresponding and senior authors for information on cancer events. RCTs were excluded if information on cancer events was not reported and could not be obtained from the study authors, or if the study had no cancer events in either treatment group. Observational studies were included only if cancer outcomes for sirolimus users and nonusers were reported in the article.

For each article, we extracted information on the type of study (RCT or observational study), geographic location, number of sirolimus users and nonusers, maximum length of follow-up, year of publication, whether sirolimus was used in recipients' initial immunosuppressant regimen, and the measure of association between sirolimus use and cancer risk (if provided). For the groups of sirolimus users and nonusers, we also extracted information on number of cancer events, use of other immunosuppressants, and sirolimus trough level (for sirolimus users).

Outcomes of interest were overall and type-specific incident cancers. We assessed effects on specific cancers when the cancer type was reported in at least five studies; this criterion identified NMSC, non-Hodgkin lymphoma, and cancers of the kidney, lung, and prostate. We also assessed effects on KS, given prior reports of transplant-associated KS regressing after initiation of sirolimus 10. As NMSC was overwhelmingly the most commonly reported cancer and likely a strong driver of the associations with total cancer incidence, we also assessed associations with cancer overall after excluding NMSC events.

### Statistical analyses

When incidence rates (IRs) or measures of association were not reported, we derived estimates based on provided information. To calculate IRs, the number of cancer events in each arm was divided by the follow-up time in each group. If a single person had multiple cancer diagnoses for different cancer types, all diagnoses were counted. Finally, an IR ratio (IRR) was calculated by dividing the IR in the sirolimus group by the IR in the comparison group. When standard errors or 95% confidence limits were not given, standard errors were instead calculated by the following formula:

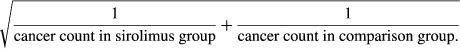


For small randomized controlled trials (SRCTs) that only had cancer events in one treatment group, results were pooled together before we calculated an overall IRR combined with the other RCTs. If the pooled SRCTs still only had cancer events in one treatment group, then a continuity correction was made by adding an artificial count of 0.1 to the cancer counts in both groups so that IRRs and standard errors would be estimable. For observational studies, the adjusted IRRs or hazard ratios and the corresponding 95% confidence intervals that were reported in the articles were used as the least biased estimate of association.

Pooled estimates were calculated for sirolimus associations with cancer incidence, using the natural log of the IRRs and the standard errors. Because of heterogeneity in the estimates for cancer overall (*P* = 0.093), a random-effects model was used in all analyses, whereas a fixed-effects model was considered in a sensitivity analysis when heterogeneity was not significant (*P* > 0.10). Publication bias was assessed using a funnel plot, in which the standard error for each study was plotted against the natural log of the IRR estimate for overall cancer.

Pooled estimates were calculated overall and stratified by study type, given the heterogeneity between RCTs and observational studies in the IRRs for overall cancer (*P* = 0.025). For RCTs, pooled estimates were also calculated separately for RCTs that compared sirolimus against cyclosporine (an immunosuppressant with possible carcinogenic properties due its disruption of DNA repair 20,21) and RCTs in which cyclosporine use was the same across treatment arms. Meta-regression was performed to evaluate additional possible modifiers of the association between sirolimus and cancer, including publication year, sirolimus trough level, follow-up time, and whether sirolimus was part of the initial immunosuppressant regimen or part of an immunosuppressant conversion.

Only two observational studies were identified, and both evaluated a large sample of U.S. kidney recipients identified through the Scientific Registry of Transplant Recipients (SRTR). As the study populations largely overlapped, results from these two studies were not pooled together. Instead, the study by Yanik et al. 17 was included in all primary analyses, because it was more recent, included estimates for individual cancer types, and had more reliable ascertainment of cancer diagnoses through linkage with cancer registries and more comprehensive information on immunosuppressant use through a linkage to pharmacy claims. In sensitivity analyses, the other observational study by Kauffman et al. 18 was substituted to evaluate whether results changed in a meaningful way.

STATA statistical software (version 13.1, StataCorp, College Station, TX, USA) was used to perform all statistical analyses.

## Results

### Literature search and included studies

**Figure 1:**
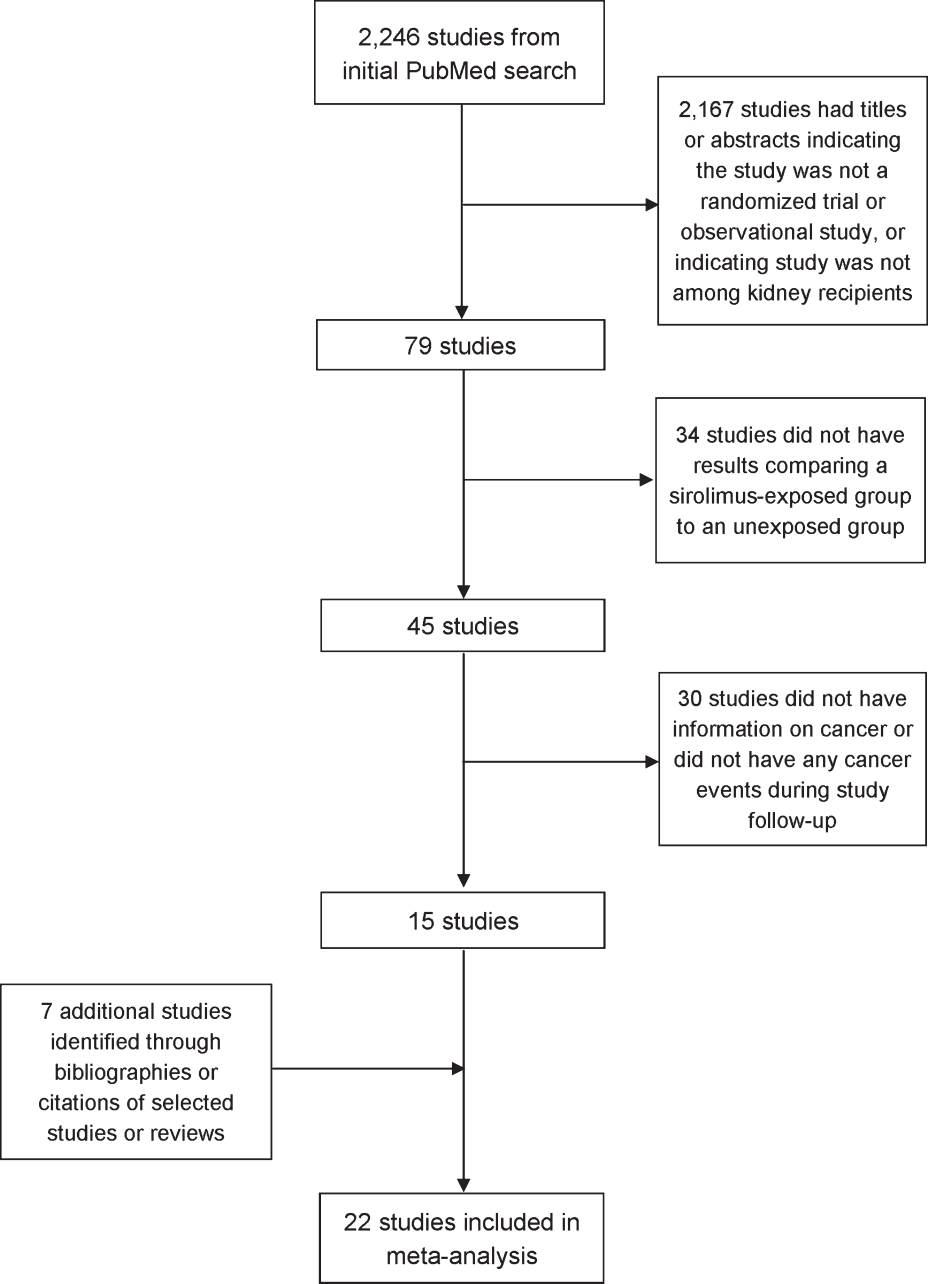
Flow chart of study selection.

We identified 2246 articles matching our search terms in PubMed. Of these, 79 articles had titles or abstracts indicating that the study was possibly a RCT or observational study of sirolimus use in kidney recipients (Fig.1). After reviewing these articles, 34 were excluded because they did not compare sirolimus-exposed recipients to unexposed recipients. Thirty articles were excluded because no malignancies were identified in the study or because information on malignancies could not be obtained. In a secondary search of bibliographies and Web of Science, we identified seven additional studies that met our inclusion criteria (Fig.1). In the final meta-analysis, 22 studies were included, of which 20 were RCTs and two were observational studies (Table1).

**Table 1:** Characteristics of included studies

First author	Publication year	Geographic location	Maximum follow-up	SRL regimen^1^	Comparison regimen^1^	Participants, *N*		Cancer events, *N*	
						SRL	Comparison	SRL	Comparison
Randomized trials									
Groth 33,2	1999	Europe	1 year	SRL and Aza	CsA and Aza	42	42	0	2
Kahan 34	2003	International	2 year	SRL and CsA	CsA and Aza/placebo	1004	291	54	20
Mendez 35	2005	United States	1 year	SRL and Tac	MMF and Tac	185	176	2	1
Watson 36	2005	Europe	1 year	SRL	CsA/Tac	19	19	2	2
Barsoum 37,2	2007	Egypt	2 years	SRL and CsA/MMF	CsA and MMF	76	37	4	0
Ekberg 38	2007	International	1 year	SRL and MMF	CsA/Tac and MMF	380	1195	9	17
Durrbach 39	2008	Europe	6 months	SRL and MMF	CsA and MMF	33	36	1	1
Glotz 40	2010	Europe	1 year	SRL and MMF	Tac and MMF	71	70	1	1
Salgo 41	2010	Europe	1 year	SRL	Non-SRL drugs	16	17	1	8
Alberú 42	2011	International	2 year	SRL, MMF, and Aza	CsA/Tac and MMF/Aza	555	275	22	34
Flechner 43	2011	International	2 year	SRL, Tac, and MMF	Tac and MMF	304	139	12	6
Lebranchu 44	2011	Europe	4 year	SRL	CsA	77	85	6	10
Campbell 31	2012	International	2 year	SRL	Non-SRL drugs	39	47	22	38
Euvrard 22	2012	Europe	2 year	SRL	CsA/Tac	64	56	23	34
Guba 45,2	2012	Europe	3 year	SRL and MMF	CsA and MMF	69	71	0	5
Lebranchu 46	2012	Europe	5 year	SRL and MMF	CsA and MMF	63	68	4	10
Chhabra 47	2013	United States	2 year	SRL and MMF	Tac and MMF	123	64	7	5
Hoogendijk-van der Akker 48	2013	Europe	2 year	SRL	Non-SRL drugs	74	81	–3	–3
Silva 49,2	2013	South America	2 year	SRL and MPS	Tac and MPS	97	107	0	2
Soleimani 50,2	2013	Asia	5 year	SRL	CsA	29	59	0	4
Observational studies									
Yanik 17	2014	United States	14 year	SRL	Non-SRL drugs	5867	26,917	85	787
Kauffman 18	2005	United States	3 years	SRL	Non-SRL drugs	2825	30,424	17	552

SRL, sirolimus; CsA, cyclosporine; Tac, tacrolimus; MMF, mycophenolate mofetil; Aza, azathioprine; MPS, mycophenolate sodium; SRCTs, small randomized controlled trials.

1 In the immunosuppressant regimen descriptions, “/” indicates a choice in the regimen, whereas “and” indicates that both immunosuppressant medications were used in the regimen.

2 These trials were combined in forest plots as SRCTs.

3 The Hoogendijk-van der Akker et al. study reported the hazard ratio relating sirolimus and nonmelanoma skin cancer risk, but did not report total cancer counts in each treatment arm.

The 20 RCTs were conducted in Europe, North America, South America, Asia, Africa, and Australia. The size of RCTs ranged from 33 recipients in the smallest trial to 1575 in the largest multi-site trial, yielding a total of 6255 recipients overall (Table1). The maximum length of follow-up ranged from 6 months to 5 years. Most RCTs were randomized with a 1:1 ratio of sirolimus users to nonusers, but one trial randomized with a 4:1 ratio, one randomized with a 1:4 ratio, and four randomized with a 2:1 ratio. The most frequently diagnosed cancer type was NMSC (reported in 15 RCTs). A number of RCTs also reported diagnoses of non-Hodgkin lymphoma (nine RCTs), kidney cancer (eight RCTs), lung cancer (eight RCTs), prostate cancer (eight RCTs), and KS (five RCTs) (Table2).

**Table 2:** Associations between sirolimus use and risk of specific cancer types

Cancer type	Number of studies	Cancer events		Results by study type^1^		*P*-value for heterogeneity between study types	Combined results^1^	
		Sirolimus group	Comparison group	Pooled incidence rate ratio (95% CI)	*P*-value		Pooled incidence rate ratio (95% CI)	*P*-value
Nonmelanoma skin cancer	15 RCT	91^2^	141^2^	0.49 (0.32–0.76)	<0.001	–	0.49 (0.32–0.76)	<0.001
	0 OS	–	–	–	–			
All other cancers	17 RCTs	79	59	1.09 (0.62–1.91)	0.762	0.486	1.06 (0.69–1.63)	0.780
	1 OS	85	787	0.94 (0.74–1.19)	0.603			
Non-Hodgkin lymphoma	9 RCT	23	6	1.78 (0.74–4.31)	0.200	0.178	1.13 (0.63–2.03)	0.682
	1 OS	7	73	0.79 (0.36–1.73)	0.556			
Kidney	8 RCT	2	15	0.19 (0.01–2.66)	0.220	0.243	0.31 (0.08–1.23)	0.096
	1 OS	9	113	0.50 (0.23–1.09)	0.081			
Lung	8 RCT	13	2	3.69 (0.51–26.94)	0.198	0.027	1.41 (0.21–9.54)	0.723
	1 OS	4	80	0.46 (0.17–1.26)	0.130			
Prostate	8 RCT	11	3	1.92 (0.24–15.12)	0.536	0.939	1.84 (0.97–3.49)	0.061
	1 OS	21	87	1.86 (1.15–3.01)	0.012			
Kaposi sarcoma	5 RCT	0	8	0.03 (0.00–14.03)	0.258	0.190	0.71 (0.02–26.91)	0.852
	1 OS	2	7	2.04 (0.40–10.35)	0.390			

RCT, randomized controlled trial; OS, observational study.

1 Random effects models were used for pooled incidence rate ratio estimates.

2 These totals do not include events from Hoogendijk-van der Akker et al. This study did not report total cancer counts in each treatment arm, but did report the hazard ratio relating sirolimus and nonmelanoma skin cancer risk, which was used in the pooled incidence rate ratio estimation.

The two observational studies based on U.S. kidney recipients in the SRTR were substantially larger than the RCTs, but had proportionately fewer sirolimus users. Yanik et al. 17 included 32,784 kidney recipients with a maximum follow-up of 14 years (Table1). During this time, 18% of kidney recipients used sirolimus at some point after transplant, as identified by pharmacy claims. Kauffman et al. 18 included 33,249 kidney recipients with a maximum follow-up of 3 years. Of these, 8.5% had sirolimus prescribed as part of their initial immunosuppressant regimen recorded in the SRTR. Both observational studies reported information on a number of cancer types, identified through linked cancer registry data in Yanik et al. and reported to the SRTR in Kauffman et al.; Yanik et al. did not include information on NMSC.

When combined, the 20 RCTs and the Yanik et al. observational study included 39,039 kidney recipients, of whom 9187 were sirolimus users. Across the studies, more than 200 NMSCs were diagnosed, and 1010 non-NMSCs were diagnosed. Non-NMSCs included 109 non-Hodgkin lymphomas, 139 kidney cancers, 99 lung cancers, 122 prostate cancers, and 17 cases of KS (Table2).

### Pooled results

**Figure 2:**
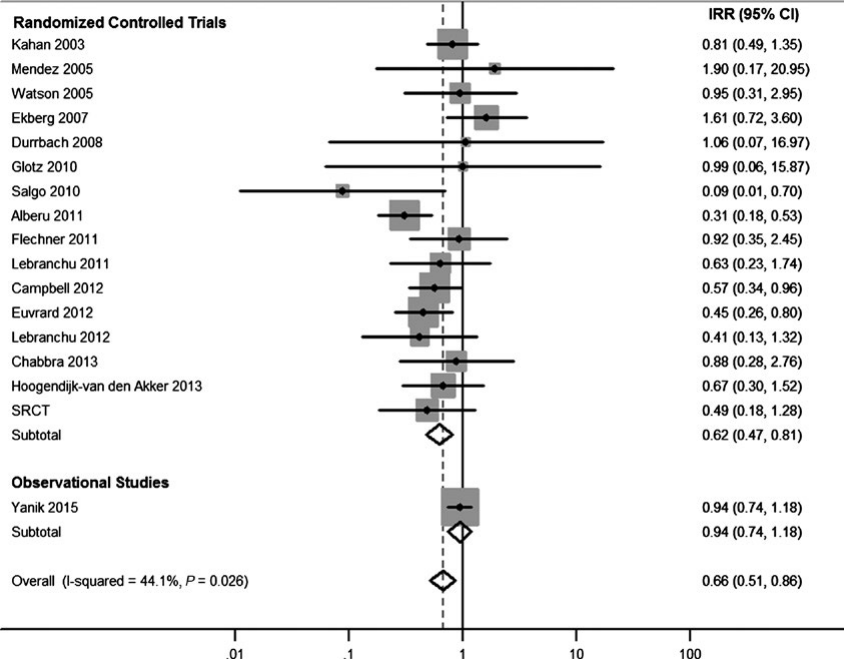
Associations of sirolimus use with overall cancer incidence, estimated in individual studies and overall. Points represent incidence rate ratio estimates for each study, whereas lines represent 95% confidence intervals. The areas of the shaded gray boxes are proportional to the inverse of the variance. Diamonds represent random effects summary estimates for randomized controlled trials, observational studies, and overall. The centers of the diamonds represent the point estimate, and the left and right points of the diamonds extend to the 95% confidence interval values. SRCT, small randomized controlled trial.

**Figure 3:**
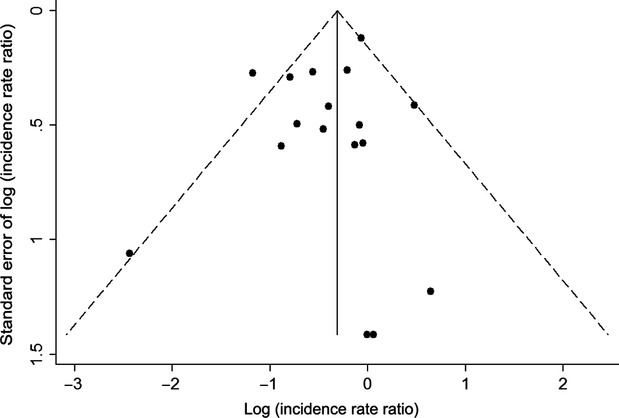
Funnel plot of included studies. Points represent the 20 randomized controlled trials and Yanik et al. observational study included in the meta-analysis. Points are plotted based on the log(incidence rate ratio) and the standard error of the log(incidence rate ratio) for the estimate of the association between sirolimus use and total cancer incidence. The solid vertical line represents the summary estimate of the log(incidence rate ratio) based on all included studies. The dashed diagonal lines represent the 95% confidence interval estimates for the summary log(incidence rate ratio) at different values of the standard error.

Overall, cancer incidence was 29% lower among sirolimus users (IRR = 0.71, 95% CI = 0.56–0.90). However, results manifested heterogeneity (*P* = 0.093), with the association differing dramatically by study type (*P* = 0.025 for heterogeneity between RCTs and Yanik et al.) (Fig.2). Among the RCTs, sirolimus use was associated with a 34% decrease in cancer incidence (IRR = 0.66, 95% CI = 0.51–0.85) and the association was not heterogeneous across RCTs (*P* = 0.148). In contrast, in Yanik et al., sirolimus use was associated with a 6% decrease in cancer incidence that was not statistically significant (IRR = 0.94, 95% CI = 0.74–1.18). Publication bias was not evident as indicated by the symmetry of the funnel plot (Fig.3).

Sirolimus use was associated with 51% lower NMSC incidence in RCTs (IRR = 0.49, 95% CI = 0.32–0.76, Table2), whereas NMSC-specific associations were not reported in either observational study. When NMSCs were excluded, there was no association between sirolimus and non-NMSC incidence in RCTs (IRR = 1.09, 95% CI = 0.62–1.91), similar to the results for total cancer incidence in Yanik et al. (Table2). Combining results from RCTs and the Yanik et al. observational study, there was no overall association with non-NMSC incidence (IRR = 1.06, 95% CI = 0.69–1.63), and results between study types were not found to be heterogeneous (*P* = 0.486).

Overall associations were not statistically significant for specific cancer types other than NMSC in random-effects analysis. However, sirolimus users had nonsignificantly lower incidence of kidney cancer in RCTs (IRR = 0.19, 95% CI = 0.01–2.66) and in the observational study (IRR = 0.50, 95% CI = 0.23–1.09; Table2). When these estimates were combined, sirolimus was associated with 69% lower kidney cancer incidence (overall IRR = 0.31, 95% CI = 0.08–1.23). In contrast, sirolimus users had significantly higher incidence of prostate cancer in the observational study (IRR = 1.86, 95% CI = 1.15–3.01), and similarly elevated incidence, though nonsignificant, in RCTs (IRR = 1.92, 95% CI = 0.24–15.12). When these results were combined, sirolimus was associated with nonsignificantly higher prostate cancer incidence (IRR = 1.84, 95% CI = 0.97–3.49). When comparing associations estimated in RCTs to associations estimated in the observational study, we did not identify heterogeneity for any cancer type, with the exception of lung cancer (*P*-heterogeneity = 0.027). Given an absence of significant heterogeneity across studies for kidney cancer (*P*-heterogeneity = 0.243) and prostate cancer (*P*-heterogeneity = 0.939), we assessed fixed-effects models, which yielded statistically significant associations with kidney cancer (IRR = 0.40, 95% CI = 0.20–0.81) and prostate cancer (IRR = 1.85, 95% CI = 1.17–2.91).

**Figure 4:**
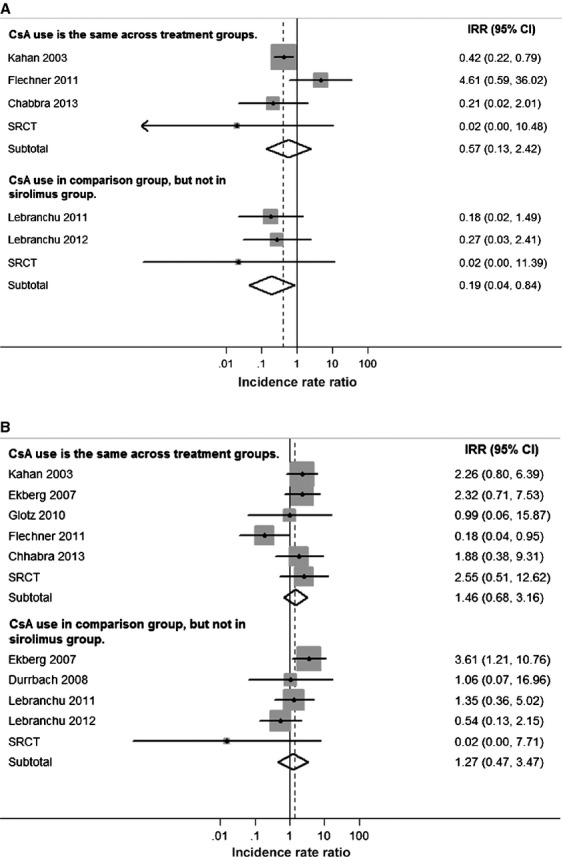
Associations of sirolimus use with nonmelanoma skin cancer and other cancers in RCTs, stratified by cyclosporine use. Associations are given for nonmelanoma skin cancers (A), and for all other cancers (B). Within each panel, results are stratified according to whether cyclosporine was used in the sirolimus arm. Points represent incidence rate ratio estimates for each study, whereas lines represent 95% confidence intervals. The areas of the shaded gray boxes are proportional to the inverse of the variance. Diamonds represent summary estimates for RCTs overall. The centers of the diamonds represent the point estimates, and the right and left points of the diamonds extend to the 95% confidence interval values. Five RCTs were excluded from (A) and three RCTs were excluded from (B) because consistent CsA use, or nonuse, was not reported in each treatment group. RCTs, randomized controlled trials; CsA, cyclosporine; SRCT, small randomized controlled trial.

Among RCTs, the association between sirolimus use and reduced NMSC incidence was much stronger in those that compared sirolimus use against cyclosporine use (IRR = 0.19, 95% CI = 0.04–0.84; Fig.4). In contrast, in RCTs where cyclosporine use was the same across treatment arms, the reduction in NMSC incidence was smaller and not significant (IRR = 0.57, 95% CI = 0.13–2.42). For non-NMSC cancers, incidence was nonsignificantly higher in sirolimus users in RCTs regardless of cyclosporine use (Fig.4).

In a meta-regression of RCT results, no additional study characteristics were associated with the IRR for NMSC (Table S1). For non-NMSCs, RCTs published in more recent calendar years and RCTs that had a higher maximum target trough level for sirolimus were likely to report more protective associations between sirolimus use and cancer incidence (Table S1). Among the five RCTs with the highest maximum target trough level (20 ng/mL), sirolimus users appeared to have lower non-NMSC incidence, although the association was not significant (IRR = 0.69, 95% CI = 0.25–1.90) (Fig. S1).

### Sensitivity analyses

The Kauffman et al. observational study reported estimates of the association of sirolimus use with total cancer incidence (including NMSCs) and with non-NMSC incidence. When Kauffman et al. was substituted for Yanik et al., the pooled IRR estimate for overall cancer incidence was lower (IRR = 0.62, 95% CI = 0.48–0.80) and significant heterogeneity was not identified between studies (*P* = 0.131). For non-NMSC cancers overall, Kauffman et al. estimated a significant protective effect (IRR = 0.45, 95% CI = 0.24–0.82). When Kauffman et al. was substituted in analyses of non-NMSC cancers, the overall pooled IRR estimate was slightly lower than when Yanik et al. was used, but still close to the null value and not statistically significant (IRR = 0.96, 95% CI = 0.56–1.65); significant heterogeneity was identified between the RCTs and Kauffman et al. (*P* = 0.014).

## Discussion

The results of this meta-analysis indicate that sirolimus use is associated with lower overall cancer incidence when compared with other immunosuppressant options for kidney recipients. Although there was heterogeneity between RCTs and the large observational study by Yanik et al. 17, this was largely explained by differences in the types of cancer events being reported. Specifically, sirolimus was associated with a 54% decrease in the incidence of NMSCs, which was only described as an outcome in the RCTs. Results for other cancers were largely similar in the RCTs and Yanik et al., and there was no evidence of an overall protective effect of sirolimus for these other cancers.

The reduction in risk for NMSCs is substantial and strongly supported by data from 15 RCTs. Sirolimus was first identified as protective against NMSCs in the Euvrard et al. RCT in 2012 22, and a protective effect was also documented in a prior meta-analysis of RCTs 19. Squamous cell skin cancers that develop in transplant recipients who are administered sirolimus manifest reduced vascularization and tumor thickness 23. In mice, sirolimus delays the growth of skin tumors induced by ultraviolet radiation 24. Nonetheless, it is notable that in our analyses, a strong protective effect appeared to be limited to trials in which sirolimus was used as an alternative to cyclosporine. Cyclosporine is classified as carcinogenic by the International Agency for Research on Cancer 25. It has been associated with increased NMSC incidence in psoriasis patients 26,27 and may encourage the development of ultraviolet radiation-related cancers by inhibiting DNA repair mechanisms 20,21. Thus, the reduced incidence of NMSC among sirolimus users may be partly driven by the withholding of cyclosporine, rather than direct antineoplastic effects of sirolimus.

In contrast, for non-NMSC malignancies overall, sirolimus use was not associated with a reduction in incidence. An absence of a protective effect was observed in both RCTs and in Yanik et al., and the association remained null when RCT and observational study results were pooled together. The other observational study by Kauffman et al. reported a protective association that was inconsistent with results from Yanik et al. and the RCTs (Kauffman IRR = 0.45, Yanik IRR = 0.94, RCT IRR = 1.05). However, Kauffman et al. relied on cancer diagnoses reported through the SRTR, which has cancer ascertainment that is less systematic and comprehensive than cancer registries, and likely less complete than within RCTs. Kauffman et al. also defined sirolimus users based only on the immunosuppressant regimen at hospital discharge 18, even though sirolimus is most frequently initiated after discharge in clinical practice due to concerns about impairment of wound healing 2.

Among the non-NMSC malignancies, the cancer type with the most evidence for a protective effect from sirolimus use was kidney cancer. Reduced incidence was seen in both RCTs and Yanik et al., with a particularly strong association identified in RCTs. Given an absence of heterogeneity, it is appropriate to consider a fixed-effect estimate for the pooled IRR, which revealed significantly lower incidence in sirolimus users. Outside of the transplant population, mTOR inhibitors are used to treat kidney cancer. For instance, the sirolimus derivative temsirolimus prolongs survival among patients with advanced stage renal cell carcinoma 12,28. These cancers are often characterized by rampant angiogenesis that is promoted by mTOR signaling, and as such are particularly vulnerable to mTOR inhibition 29,30. Given the role of the mTOR pathway in facilitating the kidney cancer growth, the reduced incidence observed in our study may reflect an effect of sirolimus in preventing small tumors from growing to a clinically detectable stage.

Prostate cancer incidence was also associated with sirolimus use, but in contrast to NMSC and kidney cancer, incidence was higher among sirolimus users. A significant association was initially reported in the observational study by Yanik et al., in which the association was driven by an increase in localized prostate cancer 17. This association has not been noted previously in individual RCTs, but none included more than five prostate cancer cases. Nonetheless, when RCTs were pooled, prostate cancer incidence was higher among sirolimus users, with an association similar to that reported by Yanik et al. Heterogeneity again was absent, and a fixed-effects estimate of the pooled IRR indicated a significantly adverse association. Despite the overall consistency of the findings, a clear biological explanation is lacking. Sirolimus could promote prostate carcinogenesis through unknown mechanisms, or it may affect serum prostate-specific antigen levels, which would in turn impact the diagnosis of prostate cancer through screening. These possibilities should be explored in further research.

For non-NMSCs overall, we found that sirolimus associations differed by study characteristics. Incidence was lower among sirolimus users when recipients were given a higher dose of sirolimus. However, even in the studies with the highest maximum trough levels of sirolimus (20 ng/mL), a significant protective effect for non-NMSCs was not found. RCTs with more recent publication years were also more likely to observe lower non-NMSC incidence among sirolimus users. Publication year could be an indicator for a variety of changes in medical care or patient characteristics that occur over calendar time, although it remains unclear how these changes would affect the association of sirolimus with cancer incidence. While we examined study level characteristics, we unfortunately could not assess individual patient characteristics, such as sirolimus blood level or individual cancer-risk factors (e.g., ultraviolet exposure, smoking).

Recently, another meta-analysis of sirolimus and cancer was conducted by Knoll et al. 14. That meta-analysis included some RCTs that we were unable to include because Knoll et al. obtained individual patient data from collaborators. However, the Knoll et al. meta-analysis was smaller than ours, did not include observational studies, and did not consider associations with individual cancer types other than NMSC. The sirolimus associations with NMSC and non-NMSC incidence were remarkably similar to the pooled associations that we identified (NMSC IRR: 0.44 in Knoll vs. 0.46 in our study; non-NMSC overall IRR: 1.05 in Knoll vs. 1.03 in our study) 14.

Our meta-analysis, including more than 39,000 kidney recipients across studies, is much larger than previous meta-analyses investigating the effect of sirolimus on cancer 13,14,19. Moreover, our review is the first to look at the effects of sirolimus on specific cancer types other than NMSC. Most of the included studies were RCTs, and we examined this group separately, so the associations with cancer we identified are unlikely to be due to unmeasured confounding in the assignment of sirolimus treatment. We were able to maximize our sample size by including observational studies, and by contacting authors of RCTs for additional information on cancer events not reported in the published articles. Notably, the results from the Yanik et al. observational study were largely consistent with the associations identified in RCTs. Publication bias does not appear to have influenced our results, as asymmetry was not detected in the funnel plot. Also, cancer incidence results probably did not influence the publication of RCTs, as most trials of sirolimus were focused on other transplant-related outcomes.

Our study has limitations. In general, cancer outcomes are rare and may take longer to develop than the typical length of a RCT. While we included one observational study with up to 14 years of follow-up, the numbers of specific cancer types other than NMSC were small. As a result, our ability to calculate precise estimates for specific cancer types was limited, though we provide the most precise estimates in the literature to date. Some RCTs had to be excluded because information on cancer outcomes could not be obtained. As cancer is an important cause of morbidity and death in kidney recipients 5, more systematic collection and reporting of this outcome would facilitate reviews in the future.

On the basis of the information from multiple RCTs and observational studies, we conclude that sirolimus use in kidney transplant recipients is associated with lower cancer risk, but this protective effect is largely limited to NMSC. Reduced NMSC incidence may be a result of the antiproliferative properties of sirolimus, or simply the removal of cyclosporine from the immunosuppressant regimen. These mechanisms are not mutually exclusive, and regardless, the results indicate that a sirolimus-based regimen may be a beneficial option for some kidney recipients, especially those with high NMSC risk (e.g., patients who have previously had multiple NMSCs) 22,31,32. Sirolimus use may also reduce risk of kidney cancer, but there was little evidence of a protective effect for other cancer types. In fact, sirolimus use was associated with higher incidence of prostate cancer. Further counterbalancing the potential benefits in reducing cancer incidence, other studies have indicated that sirolimus use may be associated with worse long-term graft function and higher mortality in kidney recipients 13–16. Therefore, in clinical practice, use of sirolimus should be carefully considered with the individual patient's medical profile along with the risk of serious medical outcomes in addition to cancer.

## Supporting Information

Table S1. Meta-regression results among randomized controlled trials. **Figure S1.** Maximum sirolimus target trough level in randomized controlled trials and associations with non-NMSC incidence.
