# Supplementary material for: Sirolimus effects on cancer incidence after kidney transplantation: a meta-analysis
Source: Cancer Med. 2015 Jun 24;4(9):1448–59. doi: 10.1002/cam4.487 (PMC4567030; doi:10.1002/cam4.487)
Supplement: Table S1. — Meta-regression results among randomized controlled trials. Figure S1. Maximum sirolimus target trough level in randomized controlled trials and associations with non-NMSC incidence. [file cam40004-1448-sd1.doc]

Supplemental Table 1: Meta-regression results among randomized controlled trials

| Study Characteristic | Unadjusted | | | Adjusted* | | |
| --- | --- | --- | --- | --- | --- | --- |
|  | Ratio of IRRs | 95% CI | P-value | Ratio of IRRs | 95% CI | P-value |
| *Non-melanoma skin cancers* | | | | | | |
| Publication year, per year | 1.04 | 0.89-1.22 | 0.538 | 1.28 | 0.94-1.75 | 0.092 |
| Maximum target trough level, per ng/mL | 1.02 | 0.89-1.16 | 0.794 | 0.99 | 0.85-1.16 | 0.934 |
| Length of follow-up, per year | 0.79 | 0.41-1.53 | 0.434 | 0.59 | 0.27-1.27 | 0.135 |
| Sirolimus conversion | 0.81 | 0.21-3.16 | 0.736 | 0.12 | 0.01-1.51 | 0.084 |
| *All other cancers* | | | | | | |
| Publication year, per year | 0.86 | 0.71-1.03 | 0.087 | 0.79 | 0.63-0.98 | 0.039 |
| Maximum target trough level, per ng/mL | 0.92 | 0.82-1.04 | 0.177 | 0.89 | 0.80-0.98 | 0.023 |
| Length of follow-up, per year | 0.81 | 0.47-1.40 | 0.412 | 0.91 | 0.57-1.47 | 0.664 |
| Sirolimus conversion | 0.87 | 0.21-3.58 | 0.834 | 1.58 | 0.44-5.76 | 0.416 |

IRR=incidence rate ratio

*The adjusted analyses include all listed study characteristics in the meta-regression model simultaneously.

Supplemental Figure 1: Maximum sirolimus target trough level in randomized controlled trials and associations with non-NMSC incidence


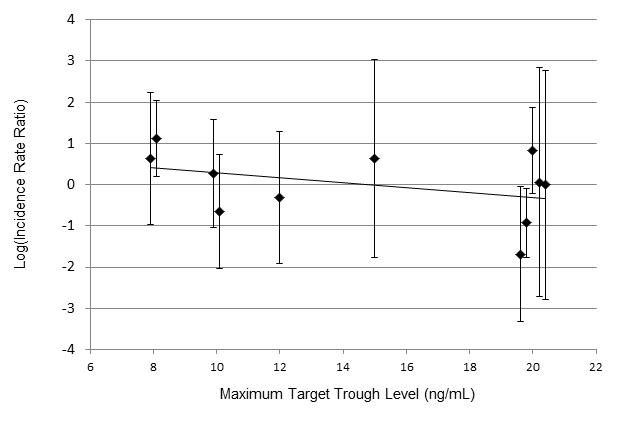


Supplemental Figure 1 Legend: For each randomized controlled trial, black diamonds represent the estimate of the natural log of the incidence rate ratio and the upper and lower error bars represent 95% confidence intervals. When more than one study had the same maximum target trough level, diamonds were slightly offset on the x-axis so that each estimate can be seen distinctly. The trend line represents the estimated association between sirolimus use and non-NMSC incidence at different trough levels based on meta-regression.

NMSC=non-melanoma skin cancer
